# Large-scale climate patterns offer preseasonal hints on the co-occurrence of heat wave and O_3_ pollution in China

**DOI:** 10.1073/pnas.2218274120

**Published:** 2023-06-20

**Authors:** Meng Gao, Fan Wang, Yihui Ding, Zhiwei Wu, Yangyang Xu, Xiao Lu, Zifa Wang, Gregory R. Carmichael, Michael B. McElroy

**Affiliations:** ^a^Department of Geography, Hong Kong Baptist University, Hong Kong SAR 999077, China; ^b^John A. Paulson School of Engineering and Applied Sciences, Harvard University, Cambridge, MA 02138; ^c^National Climate Center, Chinese Meteorological Administration, Beijing 100081, China; ^d^Department of Atmospheric and Oceanic Sciences, Fudan University, Shanghai 200438, China; ^e^Department of Atmospheric Sciences, Texas A&M University, College Station, TX 77843; ^f^School of Atmospheric Sciences, Sun Yat-sen University, Zhuhai 519082, China; ^g^State Key Laboratory of Atmospheric Boundary Layer Physics and Atmospheric Chemistry, Institute of Atmospheric Physics, Chinese Academy of Sciences, Beijing 100029, China; ^h^Department of Chemical and Biochemical Engineering, The University of Iowa, Iowa City, IA 52242

**Keywords:** air pollution, heat extremes, joint hazards, climate patterns, seasonal prediction

## Abstract

Heat waves and air pollution extremes exert compounding effects on human health and food security and may worsen under future climate change. On the basis of reconstructed daily O_3_ levels in China and meteorological reanalysis, we found that the interannual variability of the frequency of summertime co-occurrence of heat wave and O_3_ pollution in China is regulated mainly by a combination of springtime warming in the western Pacific Ocean, western Indian Ocean, and Ross Sea. These sea surface temperature anomalies impose influences on precipitation, radiation, etc., to modulate the co-occurrence, which were also confirmed with coupled chemistry–climate numerical experiments. We thus built a multivariable regression model to predict co-occurrence a season in advance, and correlation coefficient could reach 0.81 (*P* < 0.01) for the North China Plain. Our results provide useful information for the government to take actions in advance to mitigate damage from these synergistic costressors.

## Significance

Exposure to co-occurrence of air pollution and heat extremes is likely to induce amplified damages to both human health and ecosystem. This study identifies the relationship between co-occurrence of heat wave and O_3_ pollution in China and large-scale climate patterns, which offers preseasonal hints. The robustness of the findings is demonstrated with both statistical analysis and numerical coupled experiments. The results could help the government to take actions in advance to mitigate damages.

Heat waves and air pollution are two prominent threats, both of which have been reported to cause public health and ecosystem crises, particularly under rapid urbanization and global warming (1, 2). Heat waves, defined as consecutive days of excessively high atmosphere-related heat stress (3, 4), adversely influence human health by impacting respiratory and cardiovascular systems. Heat waves are linked with high O_3_ episodes that harm human health and vegetation (5–7). In warm seasons, heat waves and extreme O_3_ events often occur simultaneously due to common driving meteorological conditions, i.e., stagnant high-pressure systems that favor accumulation of heat and O_3_ precursors (8). Besides, complex chemistry–climate feedbacks through biogenic emissions (source) and uptake by plants (sink) could exacerbate co-occurrence of heat wave and O_3_ extremes (9). It is imperative to understand driving factors for the co-occurrence of heat and O_3_ extremes, as accumulating evidence suggests amplified health outcomes beyond the sum of individual effects (10–12). Analitis et al. (13) reported that the number of daily deaths during heat wave episodes was 54% higher on high O_3_ days compared with low O_3_ days.

Previous studies have linked occurrences of heat waves or O_3_ extremes, separately, with large-scale atmospheric circulation or sea surface temperature (SST) anomalies (14–20). For instance, Zhu et al. (17) demonstrated that the frequency and variability of summertime heat waves over North America was closely associated with SST anomalies in the tropical Atlantic and tropical western Pacific in spring and El Niño–Southern Oscillation phase change. Shen and Mickley (21) showed that O_3_ concentration in Eastern United States was linked with warm tropical Atlantic SST and cold northeast Pacific SST, as well as positive sea-level pressure (SLP) anomalies over central Pacific and negative SLP anomalies over the Atlantic and North America. However, the climate factors modulating the co-occurrence of heat and O_3_ extremes at a regional level remain unclear and had only been the subject of limited studies (8, 22–24).

With roughly one-sixth of the world’s population and rapid energy-intensive development, China is facing the dual challenge of air pollution and climate change (25, 26). Central and Eastern China, especially the North China Plain (NCP), experienced improved PM_2.5_ air quality over past years due to the implementation of the most stringent clean air policy, but now suffers from largest increases in summertime O_3_ exposure (27). O_3_ concentrations in the NCP enhanced at almost twice the average pace across China (28). An amplified upward trend of the joint occurrences of heat and O_3_ extremes has been identified in China over 2013 to 2020 (29). Understanding the driving climate factors for its interannual variability would contribute to long-term planning of control of costressors. Characterizing interannual variability also enables prediction which could allow sufficient time for mitigation of the interactive damages from joint exposure (21, 30–33). Previously, we demonstrated the possibility of seasonal prediction of wintertime aerosol pollution in India (34). Considering the strong linkages between O_3_ level and climate patterns, we argue here that it may also be possible to predict co-occurrence of heat waves and O_3_ pollution, potentially up to several years in advance, considering the active efforts in developing reliable seasonal (months ahead) and even longer prediction of climate variability (35).

In this study, we aim to identify leading patterns that control the spatiotemporal variability of occurrence frequency (days in a year) of joint heat wave and O_3_ pollution events (HWOP). We focus on Central and Eastern China (17.5°N to 47.5°N, 98°E to 125°E), where over 80% Chinese population reside and co-occurrences of HWOP events are prominent. Climate drivers are identified by empirical orthogonal function (EOF), which decomposes historical spatiotemporal variations of HWOP frequency that inferred with atmospheric reanalysis and reconstructed daily O_3_ datasets. Findings from statistical analyses are further supported by numerical model experiments using the state-of-the-art Community Earth System Model version 2.1.3 (CESM v2.1.3). Encouraged by the robustness of the identified teleconnections between co-occurrence events and SST anomalies, we further build a regression-based statistical model to predict summertime HWOP a season in advance, improving our capability in the management of these important health and vegetation costressors.

## Results

### Spatial Distribution and Interannual Variation of HWOP Frequency.

**Fig. 1.**
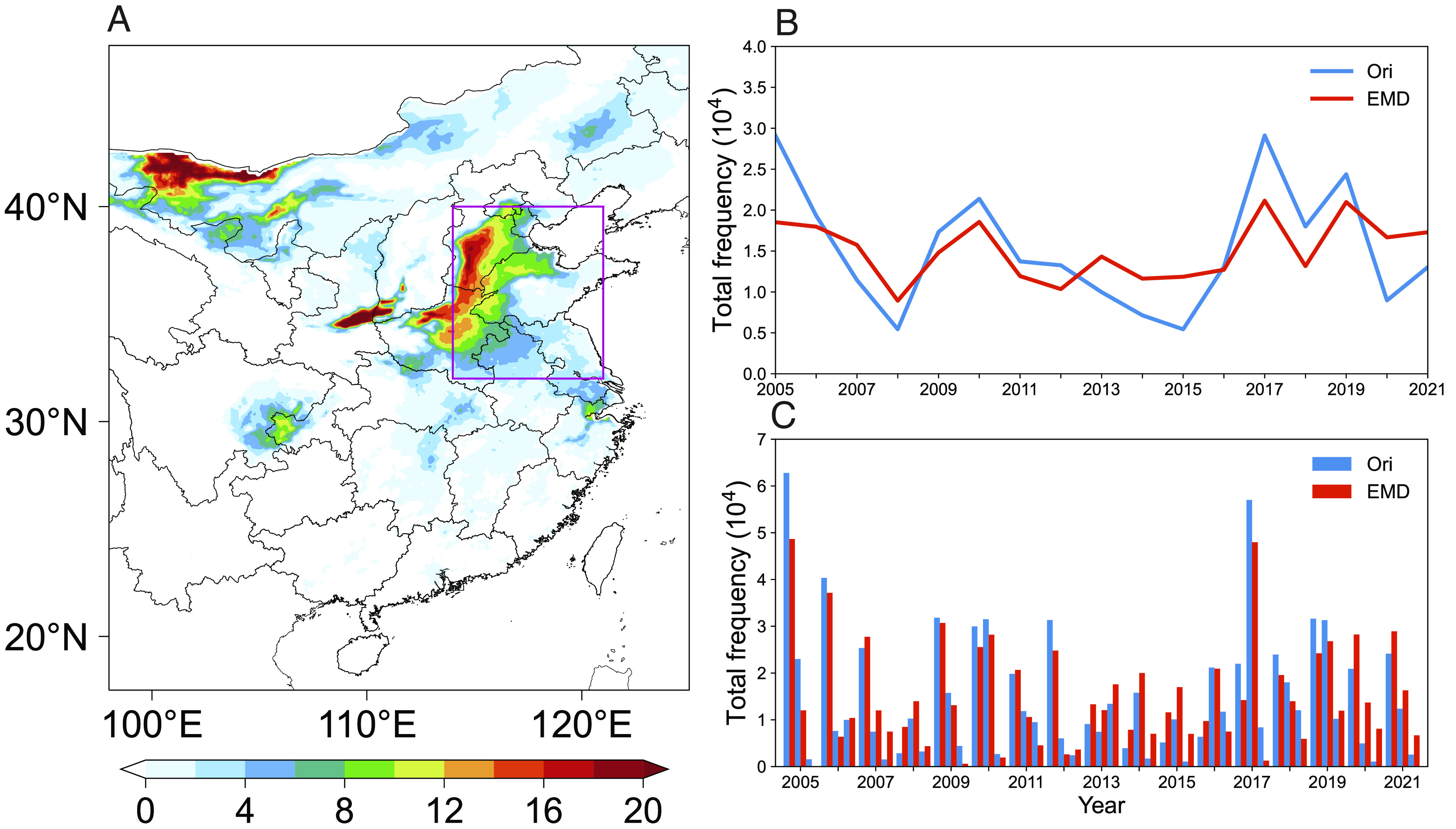
Spatial distribution and temporal variation of HWOP frequency. (*A*) Spatial distribution of mean HWOP frequency in summer (days/year) over 2005 to 2021 in Central and Eastern China. (*B*) Interannual variation of original (blue line) and detrended (red line) HWOP frequencies (# per month) in Central and Eastern China. (*C*) Intermonthly variation of original (blue bars) and detrended (red bars) HWOP frequencies (# per month) in Central and Eastern China. Pink rectangle denotes areas of the NCP, while the area inside the domain represents Central and Eastern China.

Fig. 1*A* presents the mean frequency of co-occurrence of HWOP events in summer over 2005 to 2021. We observe extensive high value (>8 d/y) in the NCP, where both O_3_ pollution and heat waves have been reported increasingly intense and frequent (36–40). Relatively lower frequencies with ~4 d/y appear in the Yangtze River Delta and Sichuan Basin. We notice that co-occurrence happens predominately (>80%) on heat wave days, while the share in all O_3_ pollution days is ~50 % (*SI Appendix*, Fig. S1). Although these proportions change with the used thresholds, we find consistently dominant role of heat waves in co-occurrence (*SI Appendix*, Fig. S2). The total frequency of HWOP for the study area (Fig. 1*A*) exhibits notable interannual variability (Fig. 1 *B* and *C*). Relatively higher frequencies occur in 2005, 2006, 2009, 2010, 2012, 2017, and 2019, while lower values appear in 2008, 2014, and 2015, partially due to relatively lower air temperature (*SI Appendix*, Fig. S3). Detrended frequencies empirical mode decomposition (EMD in Fig. 1*B*) show a different variation from the original one (ori in Fig. 1*B*) after 2013 due to the implementation of Air Pollution Prevention and Control Action Plan (26). Li et al (28) argued that anthropogenic emission contributed negatively to O_3_ anomalies over 2013 to 2016 but positively over 2017 to 2019, which is in line with our detected and removed signal of anthropogenic emissions.

## Dominant Modes of HWOP Frequency.

**Fig. 2.**
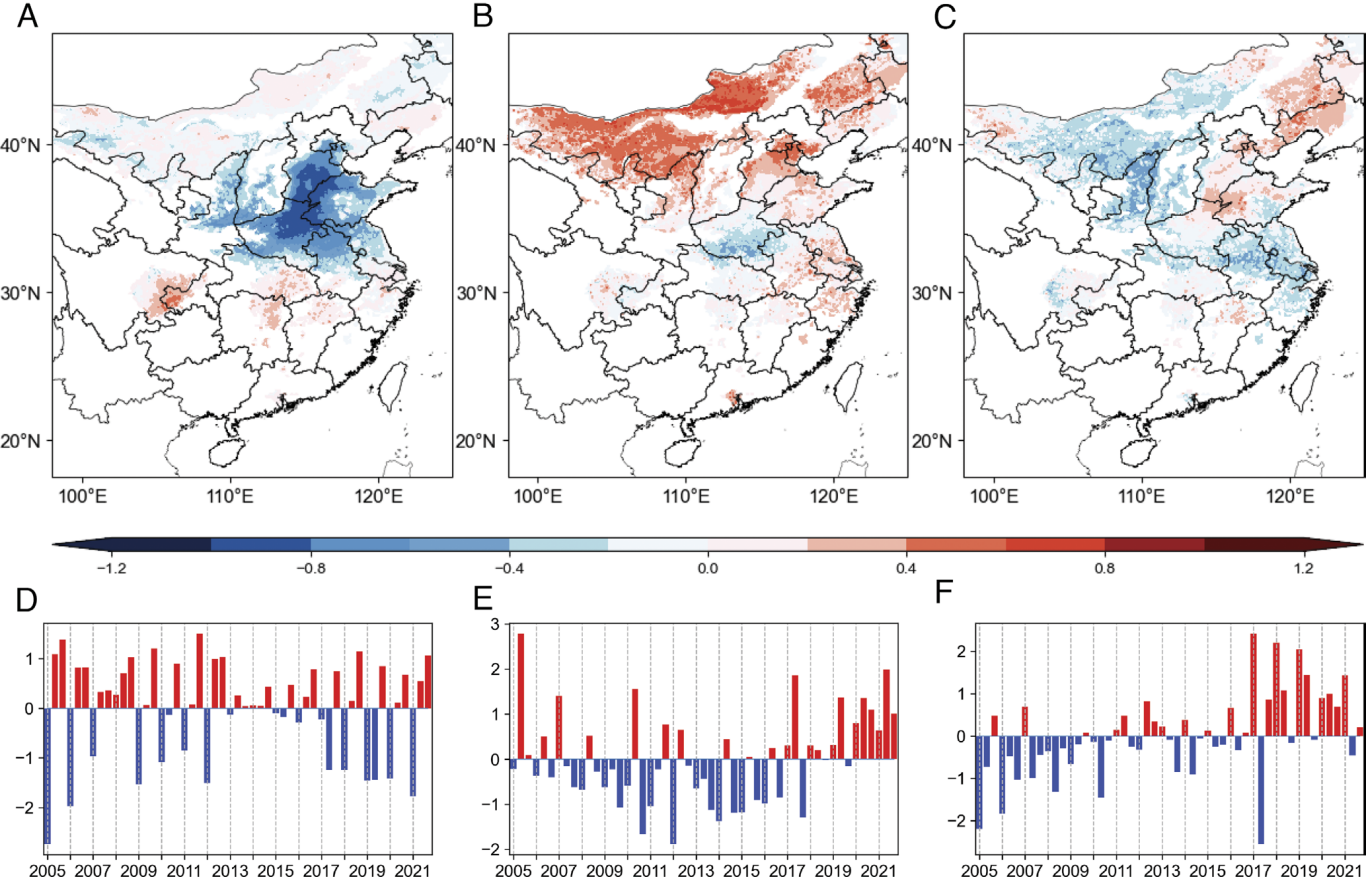
Spatial and temporal variations of the first three leading modes inferred by EOF analysis. Spatial patterns of HWOP frequency of (*A*) EOF1, (*B*) EOF2, and (*C*) EOF3. Intermonthly variation of HWOP frequency of (*D*) PC1, (*E*) PC2, and (*F*) PC3. PC values represent average over the entire domain.

EOF analysis on detrended monthly HWOP frequency over 2005 to 2021 suggests that the first three modes contribute 36%, 8%, and 6% to the total variance (*SI Appendix*, Fig. S4). The significance test of the EOF eigenvalues confirms that the first three patterns are significantly separated. Considering the lower contributions of other modes, here we focus only on the first three modes. The spatial distribution of EOF1 shows a dipole feature between northern and southern regions, with negative values in the NCP but positive values in the Yangtze River Basin (YRB) (Fig. 2*A*). The corresponding principal component (PC) of EOF1 (PC1) exhibits strong interannual variation, with lower values over 2013 to 2015, but higher values in other years (Fig. 2*D*). We also find opposite values between June and July-August, which is associated with the location of the rain belt in summer in Central and Eastern China. Rain belt is commonly located in the YRB (~ 25°N to 30°N) in June in Eastern China, yet Northern China experiences sunny and hot weather at the same time (*SI Appendix*, Fig. S5). As East Asia Summer Monsoon (EASM) marches northwards, rain season starts in Northern China, while continuous hot weather begins in the YRB (*SI Appendix*, Fig. S5) (41, 42). March of EASM and associated movement of rain belt cause the north–south shift of weather, which is represented by the toggling of positive and negative PC values. EOF2 shows positive values in most regions (Fig. 2*B*), except to the southwest of the NCP, indicating that sunny and hot weather is dominant for PC2, particularly in Beijing, Tianjin, Hebei, and Inner Mongolia. The PC of EOF2 mode (PC2) presents generally negative values before 2017 but positive values after 2017 (Fig. 2*E*). EOF3 displays a positive sensitivity extending from North China to Northeast China but negative responses in other areas (Fig. 2*C*). The variation of PC3 is similar to that of PC2, with more positive values in recent years (Fig. 2*F*).

### Warming in the Western Pacific Ocean and Excited Pacific Subtropic High Dipole.

**Fig. 3.**
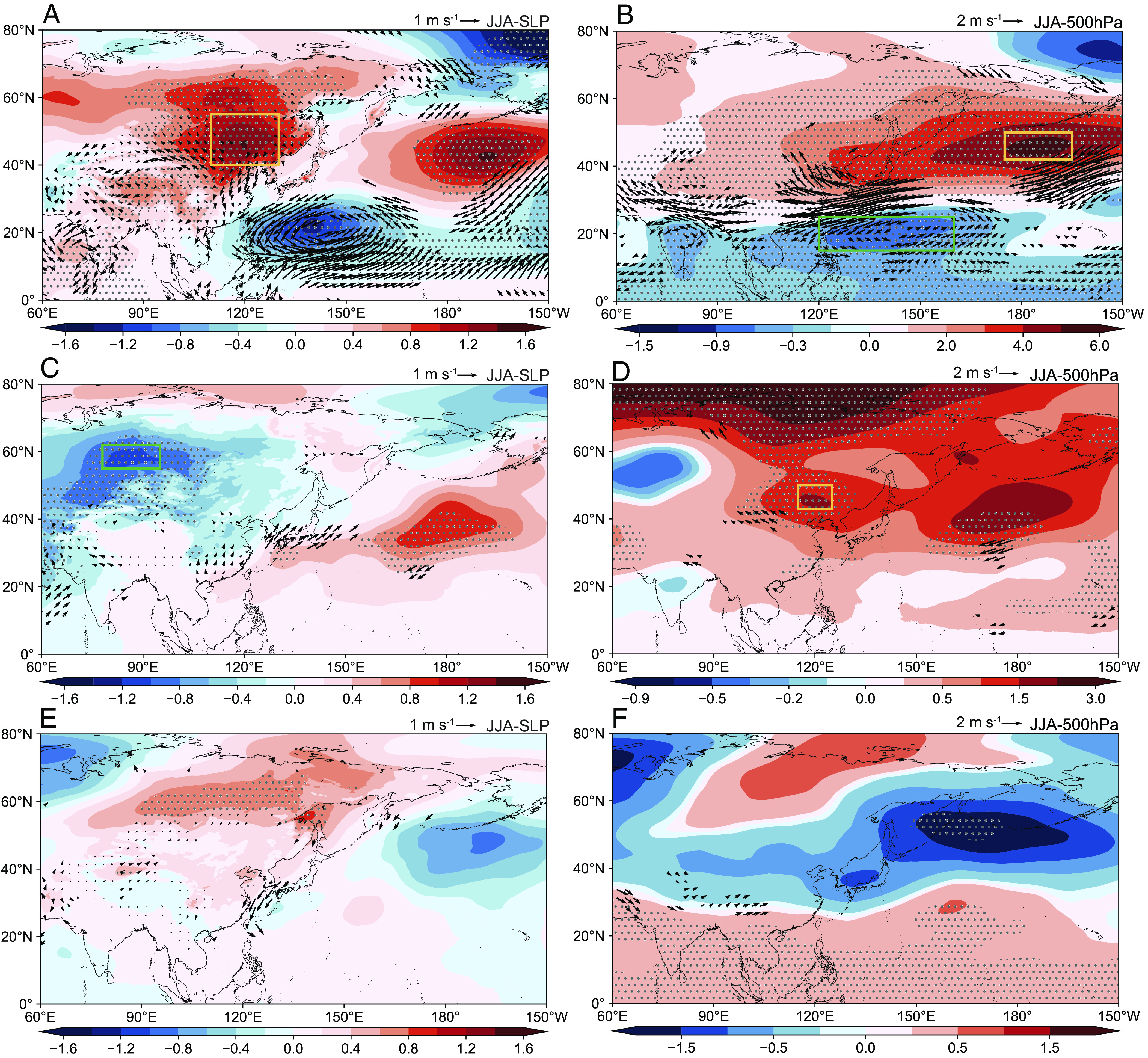
Regression of atmospheric features on leading modes. (*A*) SLP anomaly regressed on PC1; the mean anomaly of SLP within the yellow box (*A*) is defined as EA_SLP_. (*B*) Geopotential height at 500 hPa anomaly regressed on PC1; the mean anomaly of geopotential height within the yellow box (*B*) is defined as NPSH, and the mean anomaly of geopotential height within the green box (*B*) is defined as WPSH. (*C*) SLP anomaly regressed on PC2; the mean anomaly of SLP within the green box (*C*) is defined as NA_SLP_. (*D*) Geopotential height at 500 hPa anomaly regressed on PC2; the mean anomaly of geopotential height within the yellow box (*D*) is defined as EA_500_. (*E*) SLP anomaly regressed on PC3. (*F*) Geopotential height at 500 hPa anomaly regressed on PC3. Gray dots denote areas with significant correlation (*P* < 0.05).

To identify associated atmospheric patterns with the first three dominant modes, regression of anomalies of SLP, Z_500_, and wind on corresponding levels for each PC was performed. For PC1, SLP shows positive anomalies in land with the center (EA_SLP_, 40°N to 55°N, 110°E to 130°E) located in Northeastern China (yellow box in Fig. 3*A*). This enhanced pressure center is significantly associated with PC1 (r = 0.63, *P* < 0.01). Wind anomalies around the enhanced pressure center allow more cold air to flow from higher latitudes to the NCP, creating unfavorable conditions for the occurrence of high temperature. Pacific Subtropical High (PSH, also known as Hawaiian High) is the major system that affects summertime weather conditions in China, and it is conventionally measured by Z_500_ (43). Regression of PC1 on Z_500_ (Fig. 3*B*) reveals a dipole mode of PSH with weakened Western Pacific Subtropic High (WPSH, 17°N to 25°N, 120°E to 160°E) but strengthened North Pacific Subtropic High (NPSH, 42°N to 50°N, 175°E to 165°W), both of which significantly correlate with PC1 (r = 0.70 for NPSH, r = −0.53 for WPSH, *P* < 0.01). Such a spatial combination of air pressure anomalies modulates winds northward and southward of 30°N, leading to enhanced moisture transported to the NCP but weakened to the YRB. As a result, precipitation is enhanced in the NCP but decreased in the YRB (*SI Appendix*, Fig. S7*A*). Precipitation/clouds regulate downward shortwave radiation (SWD) on the ground (*SI Appendix*, Fig. S7*D*), reduce surface temperature, and suppress O_3_ formation. We thus observe reduced HWOP frequency in the NCP but enhanced HWOP frequency in the YRB. The correlation coefficient between PC1 and this dipole pattern (difference between NPSH and WPSH) is 0.72 (*P* < 0.01).

**Fig. 4.**
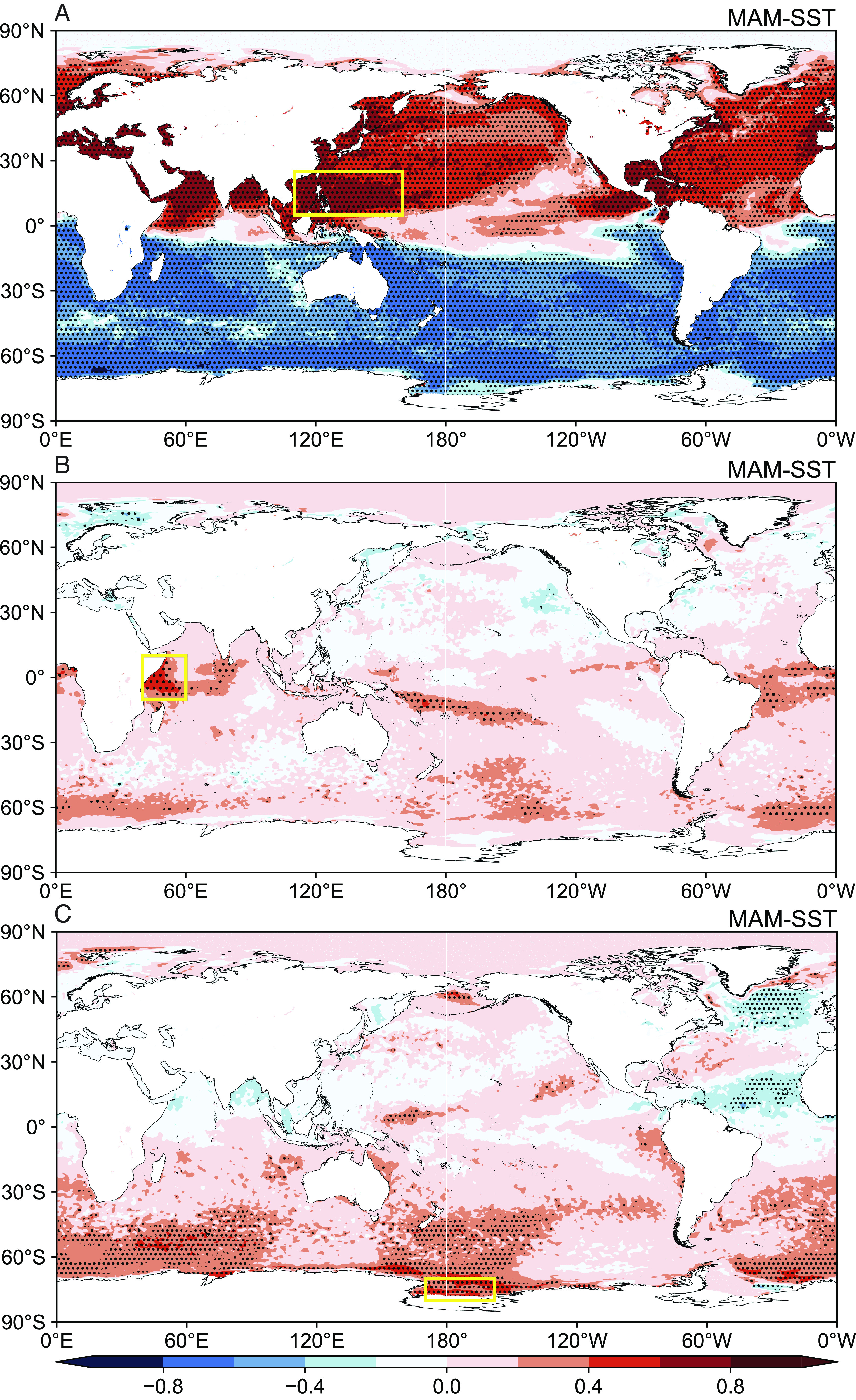
Correlations between the first three modes and springtime SST. (*A*) PC1–MAM SST correlation; the mean SST within the yellow box is defined as SST_wp_. (*B*) PC2–MAM SST correlation; the mean SST within the yellow box is defined as SST_wi_. (*C*) PC3–MAM SST correlation; the mean SST within the yellow box is defined as SST_Ross_. Positive values mean rising SST leads to increased HWOP frequency and negative values mean rising SST leads to reduced HWOP frequency. Black dots denote areas with significant correlation (*P* < 0.05).

The correlation map for spring SST anomalies resembles that for summer (*SI Appendix*, Fig. S6), suggesting that springtime SST could offer possibility of seasonal prediction. As shown in Fig. 4*A*, SST anomalies positively correlate with PC1 in the Northern hemisphere (Fig. 4*A*), especially in the western Pacific Ocean. We define SST_wp_ as the SST of the western Pacific Ocean (5°N to 25°N, 110°E to 160°E) region and find a strong connection between springtime SST_wp_ and PC1-associated summertime atmospheric patterns (0.64, 0.71, −0.49, and 0.72 for EA_SLP_, NPSH, WPSH, and NPSH-WPSH, respectively; *P* < 0.01). Regression of SST in spring and summer on PCs (*SI Appendix*, Fig. S8) reveals the process of how springtime SST anomalies affect atmospheric patterns and HWOP frequency in summer. The SST anomalies in spring mainly occur in the low latitudes of the western Pacific Ocean (*SI Appendix*, Fig. S8*A*). With the northward movement of the direct solar point and under the influence of the northward current, the SST anomalies in summer appear in the high latitudes of the Pacific Ocean (*SI Appendix*, Fig. S8*B*). Warm SST in the northern Pacific Ocean enhances anticyclonic circulation anomalies there and causes easterly winds from the North Pacific to the NCP (*SI Appendix*, Fig. S9*A*), which provides abundant moisture for precipitation (44). Composite differences confirm that warming SST of the western Pacific Ocean during March, April, and May (MAM) contributes positively to higher HWOP frequency in summer (*SI Appendix*, Fig. S10).

### Warming in the Western Indian Ocean and Associated Northward WPSH.

For PC2, SLP exhibits negative values in the middle and high latitudes of the Eurasian continent, with the center (NA_SLP_, 55°N to 62°N, 78°E to 95°E) located in western Siberia (Fig. 3*C*). PSH is enhanced over the ocean and positive values extend westward (Fig. 3*C*). An intensified pressure center (EA_500_, 43°N to 50°N, 113°E to 125°E) appears in Northeastern China, which can be considered as the northward shift of the WPSH (Fig. 3*D*). As a result, Central and Eastern China, especially northern regions, are under the control of a high-pressure system, which creates favorable conditions (e.g., sunny weather and low wind speeds) for accumulation of heat and O_3_ precursors (45). Both weakened center of SLP and intensified center of Z_500_ correlate with PC2, with correlation coefficients of –0.44 and 0.39 (*P* < 0.05), respectively.

We find that SST in the western Indian Ocean (40°E to 60°E, 10°S to 10°N) is associated with PC2 (Fig. 4*B*), and we define SST_wi_ as the average SST within this region. The correlation coefficient between SST_wi_ and PC2 is 0.42 (*P* < 0.01). The Indian Ocean Dipole (IOD) is an irregular oscillation of SSTs and related atmospheric circulation in the Indian Ocean, and the strength of IOD is commonly represented by the difference in SST between the west (50°E to 70°E, 10°S to 10°N) and southeast (90°E to 110°E, 10°S to 0°) Indian Ocean (the dipole mode index, DMI). Both DMI and SST_wi_ are highly correlated with atmospheric patterns (i.e., 0.60 and 0.63 for EA_500_, respectively; *P* < 0.01), suggesting potentially important influences on HWOP in Central and Eastern China. The IOD is able to stimulate easterly acceleration over the tropical Indian Ocean (46), which may enhance the crossequatorial flows (CEFs). Anomalous CEFs increase the westerly flow over the eastern Indian Ocean and western Pacific (*SI Appendix*, Fig. S9*B*) and further enhance the anticyclonic anomaly circulation from North China to Japan (Fig. 3*E*), forming the northward extremity of the WPSH (47). Although the found SST pattern in the Indian Ocean is not a typical IOD mode, the intensified DMI by increased SST_wi_ could exert similar influences on atmospheric circulation anomalies as IOD.

### Warming in the Ross Sea and Associated Southward WPSH.

For PC3, SLP shows an increasing tendency over land with higher values located on ~60°N (Fig. 3*E*). Pressure exhibits opposite patterns around 30°N, with positive values in the South but negative values in the North (Fig. 3*F*). This is associated with the southward shift of the WPSH. Southward WPSH may weaken East Asian monsoon and reduce moisture transport to northern regions. As a result, we observe significantly increased precipitation/decreased SWD in the middle regions of Central and Eastern China but decreased precipitation/increased SWD in Northeast China (*SI Appendix*, Fig. S7*E*). This is consistent with the spatial distribution of PC3 that the signal is negative in most areas of Central and Eastern China but positive in the northeastern regions (Fig. 2*C*). Areas with significant correlation between PC3 and springtime SST are located mainly in the mid-high latitudes of the Southern hemisphere (Fig. 4*C*). Consistently, Ledley and Huang (48) reported a statistically significant relationship between Ross Sea warming and equatorial ocean warming. We select SST of the Ross Sea (SST_Ross_, 70S to 80S, 158W to 170E) as the signal of ocean warming of the Southern hemisphere. The correlation coefficient between SST_Ross_ and PC3 reaches 0.48 (*P* < 0.01). Springtime SST anomalies propagate northward from the Antarctic region, resulting in widespread increases in SST throughout the Southern hemisphere and the Indian Ocean in summer (*SI Appendix*, Fig. S8*F*). SST anomalies over these regions enhance westerly wind at 30°N (*SI Appendix*, Fig. S9*C*) and induce a weakened WPSH in East Asia, which is unfavorable for moisture transport from the low-latitude regions to North China (49).

### Numerical Model Verification Using CESM Experiments.

**Fig. 5.**
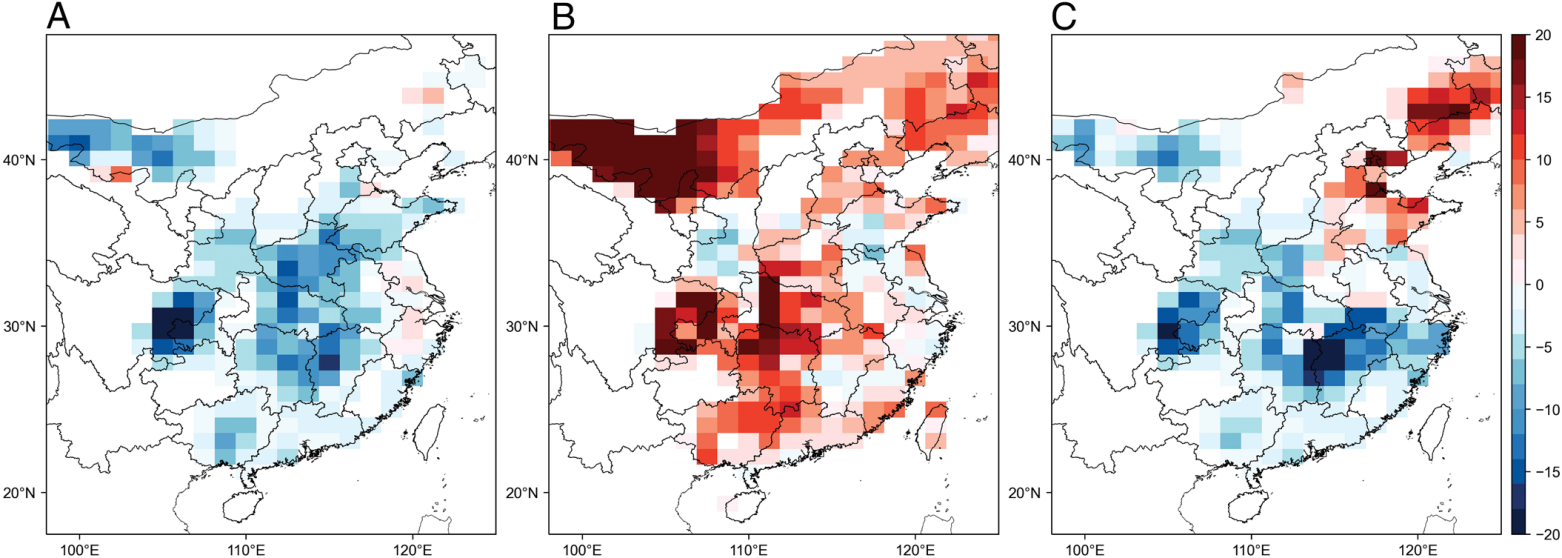
CESM-simulated responses of HWOP frequency. CESM-simulated responses of HWOP frequency to springtime (*A*) SST_wp_ anomaly, (*B*) SST_wi_ anomaly, and (*C*) SST_Ross_ anomaly.

Considering the model deficiency in capturing long-term observed variations of SST, we imposed these SST anomalies in the CESM2 model to verify proposed influences of warming in the western Pacific Ocean, western Indian Ocean, and Ross Sea. As shown in Fig. 5, simulated responses of HWOP to SST anomalies are generally consistent with those from EOF decomposition, confirming the observed relationship between SST anomalies and HWOP frequency in Central and Eastern China. SST_wp_ anomalies enhance easterly wind in regions around 30°N and increase moisture transport to the NCP, leading to suppressed SWD (*SI Appendix*, Fig. S11*A*), reduced air temperature, and lower O_3_ (*SI Appendix*, Fig. S11 *D* and *G*). We imposed only changes in SSTs of the western Pacific Ocean (5°N to 25°N, 110°E to 160°E, Fig. 4*A*) in the simulation, yet warming or cooling associated with PC1 is widespread. This causes strengthened NPSH located to the west in the simulation (*SI Appendix*, Fig. S12). As a result, anomalous easterly wind over China partially moves southward, causing insufficient moisture transport to the NCP but increased moisture transport to the southern region (*SI Appendix*, Fig. S12*A*). Although we find shifted responses to some extent in the southern region, our simulation results confirm that warming in the west Pacific Ocean could excite Pacific Subtropic High dipole and lead to corresponding responses of HWOP frequency. In contrast, SST_wi_ anomalies weaken monsoon in East Asia, resulting in warming land and increasing O_3_ concentration, especially in the NCP (*SI Appendix*, Fig. S11 *E* and *H*), which is in line with the spatially positive values of EOF2 in Fig. 2*B*. SST_Ross_ anomalies excite an anticyclone enhancement in the NCP, which are favorable for warm and dry conditions (*SI Appendix*, Fig. S11 *F* and *I*).

### Statistical Seasonal Prediction.

**Fig. 6.**
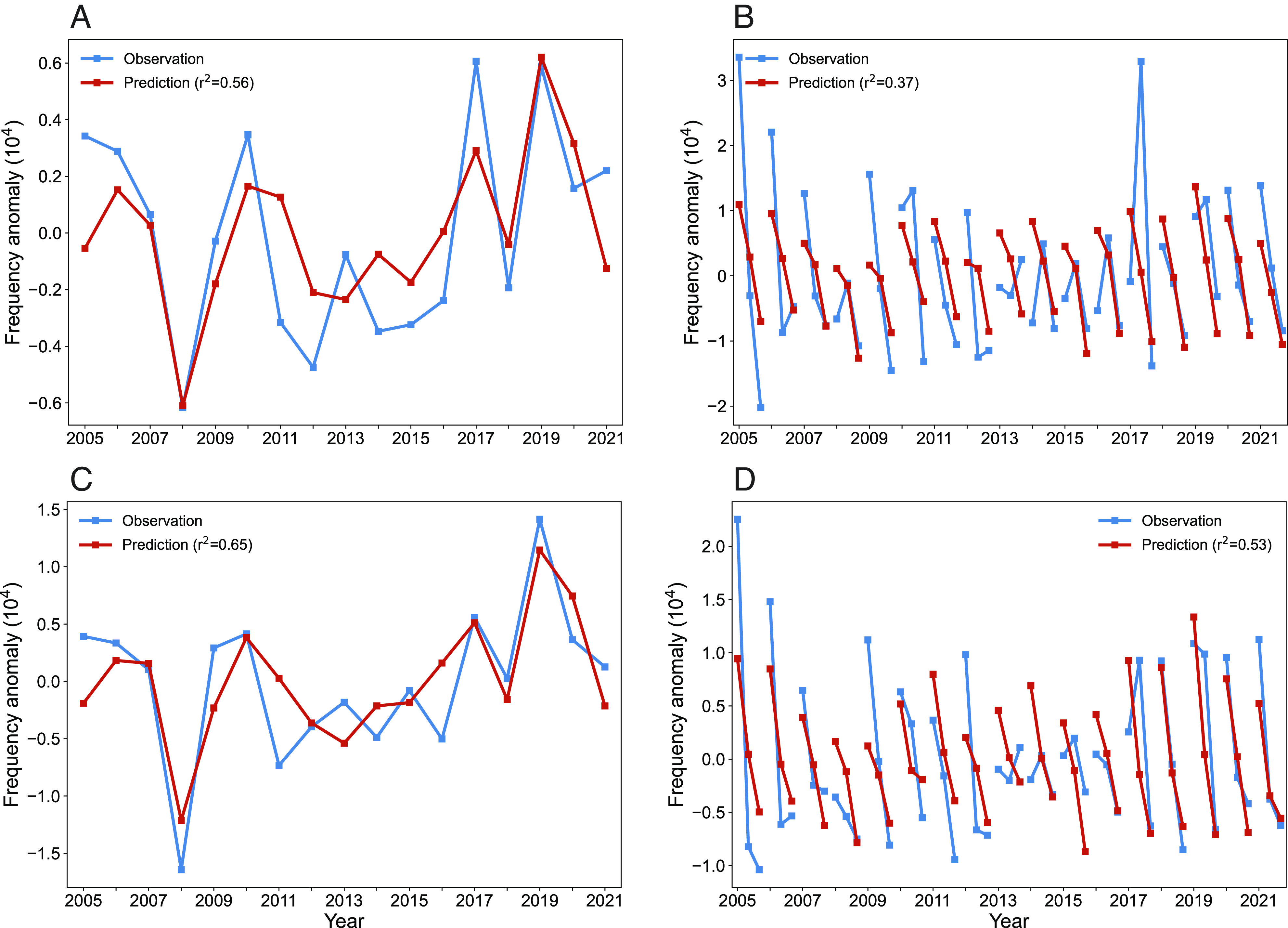
Multivariable regression modeling. Time series of annual (*A*) and monthly (*B*) HWOP frequency anomaly in Central and Eastern China. Time series of annual (*C*) and monthly (*D*) HWOP frequency anomaly over the NCP. Observations are represented in blue. Predictions using the MLR model are indicated in red.

Based on these lagged influences of SSTs on HWOP, we developed an multiple linear regression (MLR) model incorporating springtime SST_wp_, SST_wi_, DMI, and SST_Ross_ through the following equation:

$$\begin{array}{c} \text{HWOP}=a_{0}+a_{1}{\mathrm{SST}}_{\mathrm{wp}}+a_{2}{\mathrm{SST}}_{\mathrm{wi}}+a_{3}\mathrm{DMI}+a_{4}{\mathrm{SST}}_{\mathrm{Ross}}, \end{array}$$

where a_0_, a_1_, a_2_, a_3_, and a_4_ denote the coefficients that determined through the multivariable regression procedure. To assess the significance of statistical models with respect to effectiveness and overfitting potential, models constructed using 15 combinations of these four predictors were crosscompared using the Akaike information criterion [AIC, Sakamoto et al. (50)]. We find that the model incorporating all the four predictors performs best with the lowest AIC value of 338.30 (*SI Appendix*, Table S1). Our built model is able to foresee HWOP frequency a season ahead, and determination coefficients reach 0.56 (*P* < 0.01) for prediction in Central and Eastern China and 0.65 (*P* < 0.01) for the NCP region (Fig. 6 *A* and *C*). We also construct predictions at monthly (June, July, and August, JJA) scale, and reasonably moderate performances are also yielded (Fig. 6 *B* and *D*) with r^2^ values of 0.37 (*P* < 0.01) and 0.53 (*P* < 0.01), respectively.

## Discussion

The link between climate patterns and heat waves or O_3_ pollution in China has been well documented, yet the understanding of their joint occurrence has received less attention. In this study, we identified three leading modes of spatiotemporal distribution of HWOP frequency in China. We linked these three modes with Pacific Subtropic High dipole, northward WPSH, and southward WPSH, through which precipitation and SWD are modulated (*SI Appendix*, Fig. S7) to affect HWOP frequency. Although the formation of O_3_ pollution can be affected by biogenic emissions of VOCs (BVOCs) (9) and drought conditions in the previous year are likely to suppress BVOCs (*SI Appendix*, Figs. S13 and S14), HWOP frequency is mainly controlled by precipitation in the same year considering a larger correlation between HWOP and soil water for the same year (*SI Appendix*, Figs. S15 and S16). Further, we recognized the important roles of springtime SST anomalies in the western Pacific Ocean, the Indian Ocean, and the Ross Sea. The SST influences on HWOP were examined with both statistical analysis and SST-driven numerical simulations. A statistical model was also established accordingly to foresee co-occurrence of heat and O_3_ extremes at least a season in advance.

Using 15 y of surface observations, Schnell and Prather (8) revealed features of co-occurrence of temperature, O_3_, and particulate matter extremes in the United States. Despite that the compounding effects of heat and O_3_ extremes on vulnerable population groups have been realized, the characteristics and predictive potential have not been well understood in China. One major reason is the lack of long-term daily observations of ground-level O_3_ concentrations. We overcome such limitation by reconstructing a daily O_3_ dataset using a sophisticated machine learning approach. Our results are also affected by our definition of extremes based on absolute values, and we examined how alternative absolute thresholds and percentile thresholds would make a difference. As shown in *SI Appendix*, Fig. S17, using alternative absolute thresholds yielded similar interannual variations. Although using percentile thresholds changes spatial distribution of HWOP frequency, relatively consistent interannual variations were found for our concerned NCP region where residents are exposed to high co-occurrence of heat waves and O_3_ pollution.

Under a warming climate, heat waves and O_3_ pollution are projected to become more intense over most global land areas with greater maximum temperatures until the end of this century (22). The summer of 2022 witnessed record-breaking heat waves in places around the world, including megacities, where local emissions are substantial to form O_3_ pollution. How to avoid the harm by these synergistic costressors is a challenge, and our results have important operational implications. Daily global SSTs observed by satellite and in situ platforms, such as buoys and ships, are being offered continuously by multiple agencies, such as National Oceanic and Atmospheric Administration. These operational data in spring could be substituted into the statistical model we built to predict potential HWOP extremes in the following summer. This allows a season or several months in advance for Ministry of Ecology and Environment of China (MEE) to take actions. If predictions suggest more HWOP extremes in the coming summer or months, the MEE could issue warnings in their operational services so that agriculture or other related sectors or people who are sensitive to these extremes could be prepared ahead. The MEE could also optimize their management plan for air pollutants and greenhouse gases to face the incoming extremes by setting more stringent control targets or organizing sources of electricity generation.

The influences of rapidly changing anthropogenic emissions and other factors can be further considered in the future to improve the capability of our prediction. We also noticed from analysis that heat waves play more decisive roles in the co-occurrence. In addition to reducing emissions of air pollutants to improve air quality, controlling emissions of greenhouse gases to slow down or curb warming is also vital to reduced exposure to co-occurrence of costressors. The influences of emission pathways on future changes in the joint occurrence of heat waves and O_3_ pollution are not discussed in this study, which deserves future explorations. Previously, carbon reduction and air pollution control were usually considered separately, while comitigation of heat waves and air pollution requires a synergy to address this challenge.

## Materials and Methods

### Daily O_3_ Datasets.

The gridded daily O_3_ concentrations in China [Zhou et al. (51); freely available at https://zenodo.org/record/6507706#.Yo8hKujP13g] cover the period of 2005 to 2021 at a spatial resolution of 0.1° × 0.1°. This O_3_ dataset was reconstructed with an eXtreme Gradient Boosting (XGBoost) model that integrated high-resolution meteorological data, satellite retrievals of trace gases, etc., and both crossvalidation and independent validation with historical observations of O_3_ in China confirmed the accuracy. We used this daily dataset to identify high O_3_ days using the ambient air quality standards (GB 3095-2012) released by the MEE, which defined polluted days as when daily maximum 8 h average (MDA8) O_3_ concentrations exceeded 160 μg m^−3^ (52).

### Meteorological Reanalysis.

We used hourly gridded 2m air temperature (T_2m_) at the same spatial resolution of 0.1° × 0.1° and over the same period of 2005 to 2021 from the European Centre for Medium-Range Weather Forecasts (ECMWF) ERA5-Land dataset (53, 54) to determine occurrences of heat waves. Following the standard set by the China Meteorological Administration, we defined occurrences of heat waves when daily maximum T_2m_ exceeds 35 °C for at least three consecutive days (55). After days of high O_3_ and heat waves were defined separately, we calculated the frequency (number of days) of their co-occurrences (both heat waves and O_3_ pollution occurred on the same day) in each month of summers (JJA) over 2005 to 2021. We then averaged the days of co-occurrence in each grid over 2005 to 2021 to obtain the spatial distribution of HWOP frequency (days/year) and summed up the number of co-occurrence in Central and Eastern China region to derive interannual and intermonthly variations (Fig. 1).

To understand the co-occurrence-associated climate factors, we also obtained surface variables of monthly SLP, SST, 10m u-component of wind (U_10m_), and 10m v-component of wind (V_10m_), as well as midtropospheric variables of monthly geopotential height (Z_500_), u-component of wind (U_500_), and v-component of wind (V_500_) at 500 hPa from the ECMWF ERA5 dataset (53). All of these variables were at a spatial resolution of 0.25° × 0.25°.

### Statistical Analysis.

We adopted EOF analysis to decompose spatiotemporal variations of HWOP frequency over 2005 to 2021 in Central and Eastern China. We focus on the first three modes, and EOF1, EOF2, and EOF3 were significantly separated (56). To remove the impacts of anthropogenic emissions, we detrended HWOP frequency using the EMD method for each grid. The EMD method decomposes the input spatiotemporal variation into several intrinsic mode functions (IMF) and a residue (57). Given the minimum frequency of the last IMF, we considered it as the signal of anthropogenic emissions and removed it. The signal of anthropogenic emissions includes the impacts of both the trends of O_3_ precursor emissions and changes in aerosols. It was concluded that both decreases in PM_2.5_ and unmitigated emissions of volatile organiccompounds drove the increase in O_3_ (28). Regarding the influence of aerosol loadings on O_3_ formation, changes in heterogeneous reactions were found to play a more important role than the increase in photolysis rates due to lower aerosols (28, 58). We also conducted composite analysis on SST of months when high and low HWOP frequencies occurred to confirm the role of SST. The high and low HWOP frequencies were defined as those larger than one SD. SST was deseasonalized by subtracting its respective monthly mean annual cycles at each grid point before composite.

### CESM Experiments.

CESM v2.1.3 was used to explore how HWOP responds to changes in springtime (MAM) SST patterns. The selected component set (compset) was FWHIST, the robustness of which has been validated extensively (59). FWHIST was configured with a horizontal resolution of 0.9° × 1.25° and 70 vertical layers. The Community Atmosphere Model version 6 (60) was used to simulate atmospheric physics, while the Whole Atmosphere Community Climate Model version 6 (61) was used to describe tropospheric, stratospheric, mesospheric, and lower thermospheric chemistry. The Data Ocean Geophysical Model (62) was used to provide SSTs, which allows the applications of SST anomalies for sensitive experiments. Land processes were characterized by the Community Land Model version 5 [CLM5, Lawrence et al. (63)], and other selections included the Sea Ice Model version 5 (64) for sea ice, the Model for Scale Adaptive River Transport (65) for river runoff, the Community Ice Sheet Model Version 2 (66) for land ice, and the Stub wave component for wave. Anthropogenic emissions were obtained from the Community Emissions Data System (67), while biomass-burning emissions were provided by van Marle et al. (68). Biogenic emissions were calculated online using the Model of Emissions of Gases and Aerosols from Nature version 2.1 that was incorporated in the CLM5 model (69). Corresponding with the decomposed climate modes, four sets of simulations were designed with springtime (MAM) SST, namely CESM_ctrl_, CESM_wp_, CESM_wi,_ and CESM_Ross_. CESM_ctrl_ was the control case with monthly varying climatological SST data. For CESM_wp_, CESM_wi,_ and CESM_Ross_, SST anomalies were applied, respectively, in the west Pacific Ocean, the western Indian Ocean, and the Ross Sea in Antarctica, following the values obtained from regression analysis. All of these experiments were run from January to September 2010 as SST anomaly was smallest in 2010. We evaluated the performance of CESM in simulating variations of air temperature and O_3_ concentrations. As shown in *SI Appendix*, Fig. S18, general variations of surface air temperature and O_3_ concentration were well reproduced by CESM_ctrl_. The mean fractional biases (MFBs) and the mean fractional errors (MFEs) meet the model performance criteria of within ± 60% for MFB and lower than +75% for MFE (70). Considering these biases (61, 71), the simulated results were used only to investigate the direction of the response instead of the exact magnitudes.

## Supplementary Material

Appendix 01 (PDF)

## Data Availability

Gridded daily O_3_ concentrations in China data have been deposited in Zenodo (https://zenodo.org/record/6507706#.Yo8hKujP13g) (51).
