# Supplementary material for: Large-scale climate patterns offer preseasonal hints on the co-occurrence of heat wave and O3 pollution in China
Source: Proc Natl Acad Sci U S A. 2023 Jun 20;120(26):e2218274120. doi: 10.1073/pnas.2218274120 (PMC10293814; doi:10.1073/pnas.2218274120)
Supplement: Supplementary file 1 — Appendix 01 (PDF) [file pnas.2218274120.sapp.pdf]

PNAS

Supporting Information for

Large-scale climate patterns offer pre-seasonal hints on the co-occurrence of heat wave and O_3 pollution in China.

Meng Gao, Fan Wang, Yihui Ding, Zhiwei Wu, Yangyang Xu, Xiao Lu, Zifa Wang, Gregory R. Carmichael, Michael B. McElroy

Corresponding author. Meng Gao and Zifa Wang
Email: mmgao2@hkbu.edu.hk (M.G.); zifawang@mail.iap.ac.cn (Z.F.W.)

This PDF file includes:

Figures S1 to S18
Tables S1

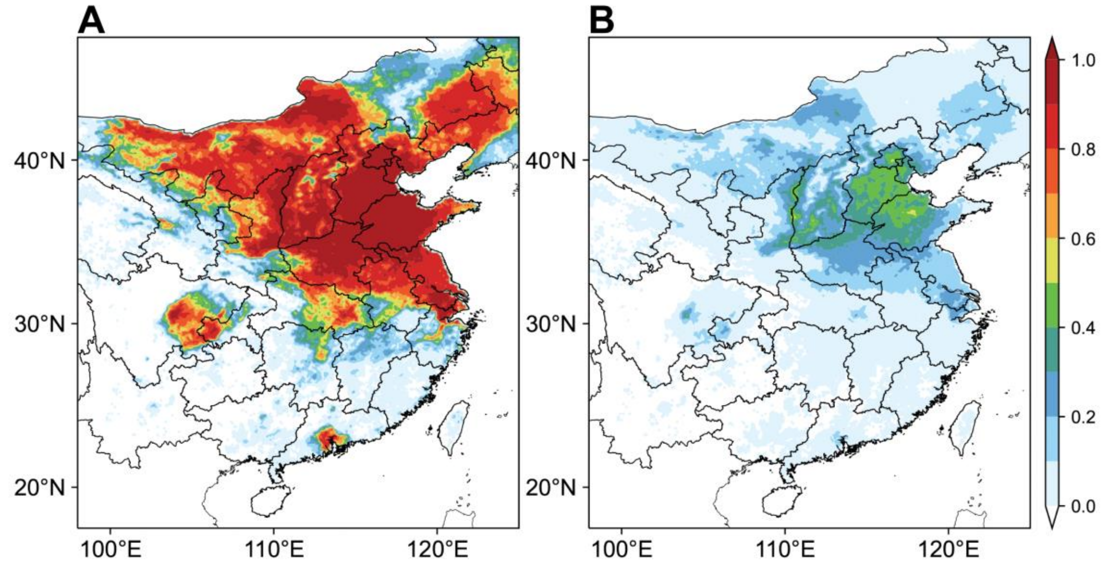

Fig. S1. Spatial distribution of proportions of co-occurrence days in all heat wave or O_3 pollution days. (A) Spatial distribution of proportions of co-occurrence days in all heat wave days; (B) Spatial distribution of proportions of co-occurrence days in all O_3 pollution days.

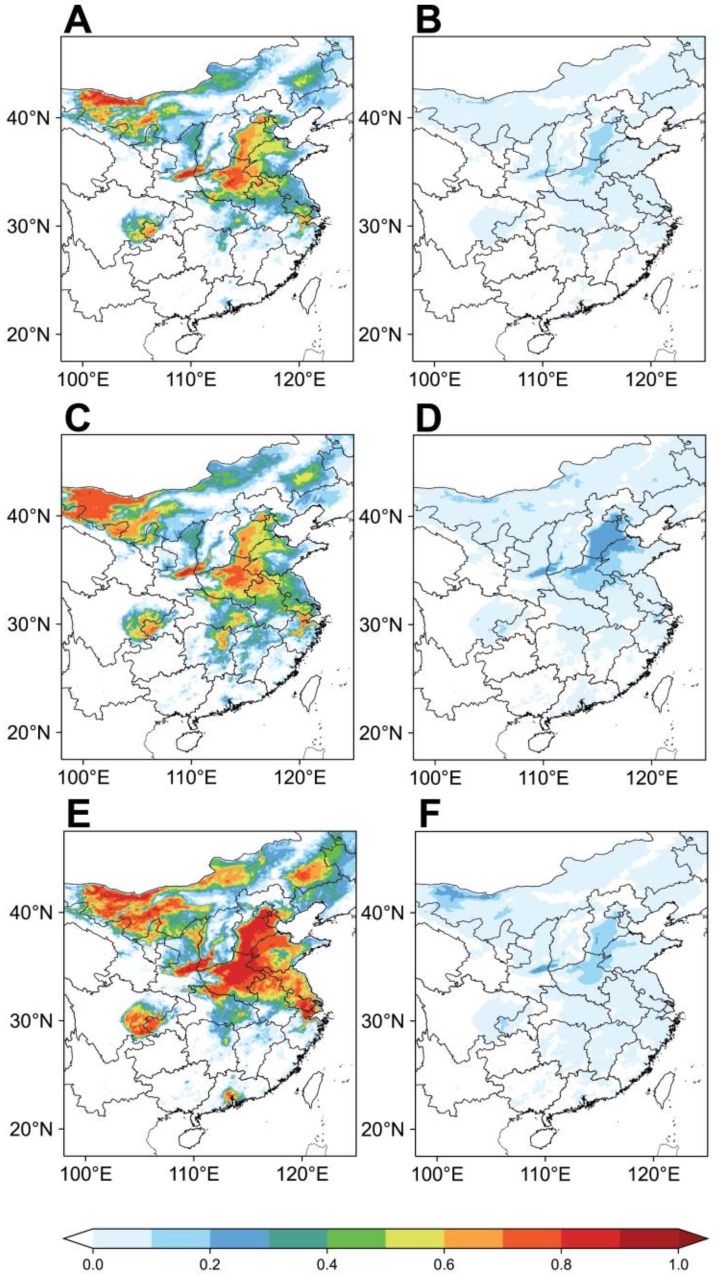

Fig. S2. Spatial distribution of proportions of co-occurrence days in all heat waves or O_3 pollution days based on different thresholds. (A, C, E) Spatial distribution of proportions of co-occurrence days in all heat waves days; (B, D, F) Spatial distribution of proportions of co-occurrence days in all O_3 pollution days; (A, B) Daily maximum T2m exceeds 35 °C for at least three consecutive days and daily MDA8 O_3 concentrations exceed 160 μg m^{-3}; (C, D) 33 °C for T2m and 160 μg m^{-3} for MDA8 O_3 concentrations; (E, F) 35 °C for T2m and 140 μg m^{-3} for MDA8 O_3 concentrations.

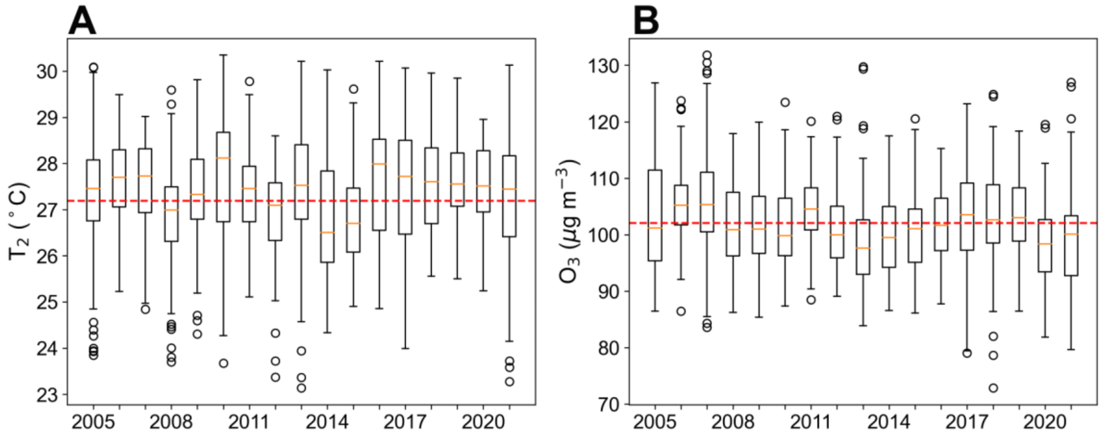

Fig. S3. Distribution of (A) daily maximum 2m air temperature (T_2) and (B) daily maximum 8 h average (MDA8) O_3 concentrations in summer over 2005-2021 in Central and Eastern China. Yellow lines within the box are median values; box chart values from the bottom to up represent the mean value minus one time of standard deviation, 25% quantile line, 75% quantile line, and the mean value plus one time of the standard deviation; hollow circles represent outliers. Red lines are average values for all years.

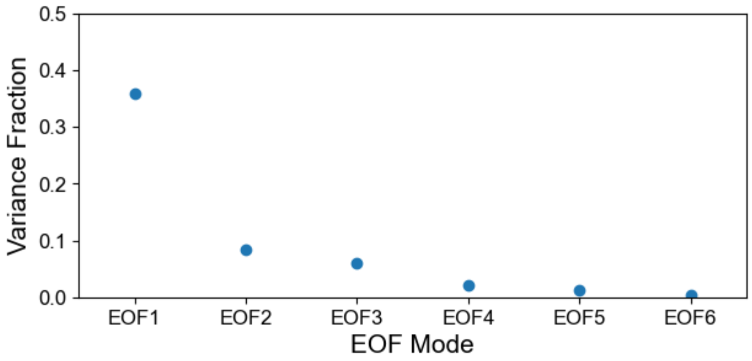

Fig. S4. Variance Fraction explained by the first six EOF modes of co-occurrence frequency.

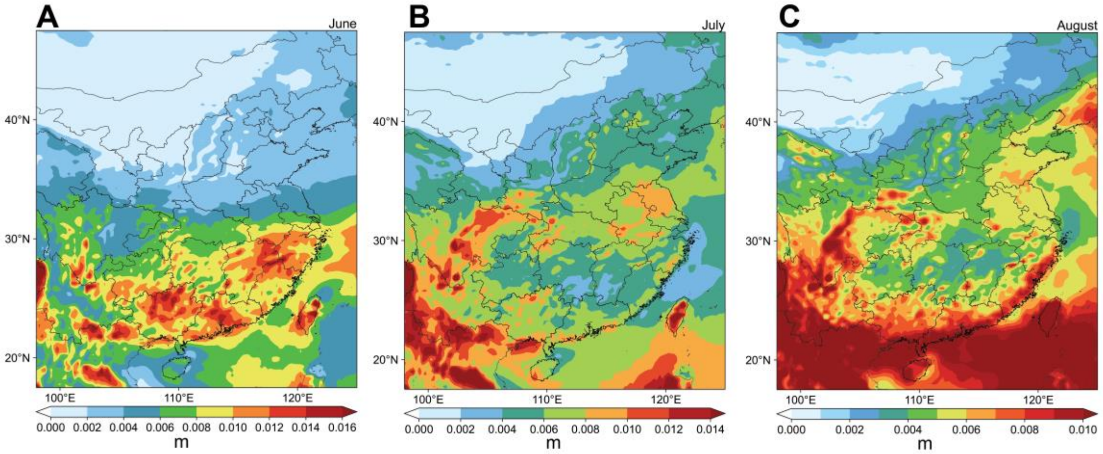

Fig. S5. Spatial distribution of total precipitation in summer inferred from ERA5. Spatial patterns of total precipitation in (A) June, (B) July, and (C) August.

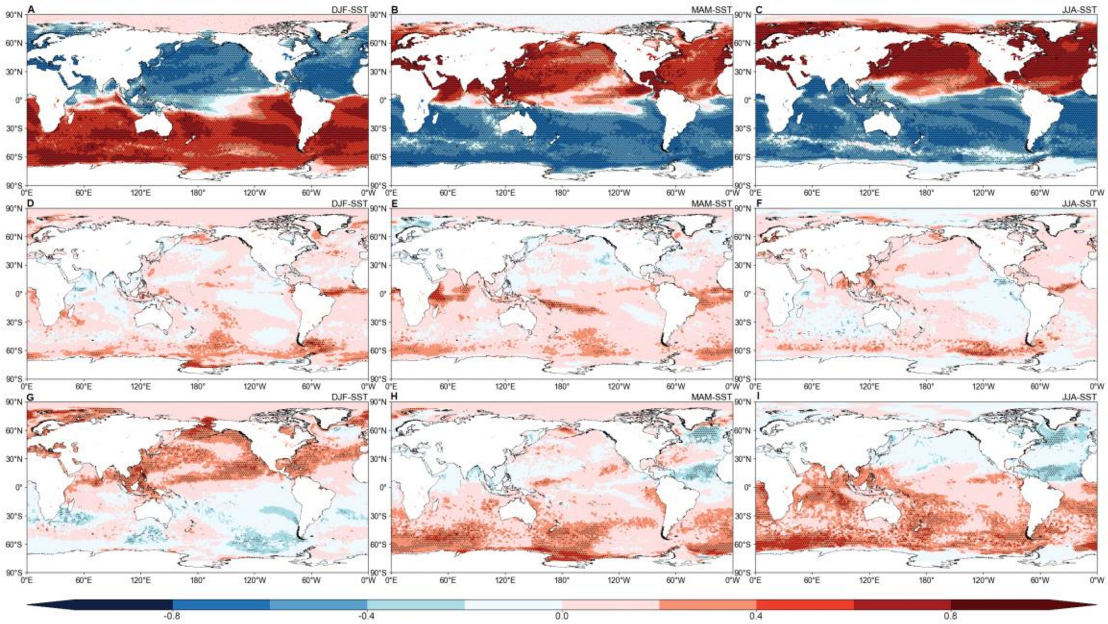

Fig. S6. Correlations between the first three modes and sea surface temperature (SST). (A) PC1-DJF SST correlation; (B) PC1-MAM SST correlation; (C) PC1-JJA SST correlation; (D) PC2-DJF SST correlation; (E) PC2-MAM SST correlation; (F) PC2-JJA SST correlation; (G) PC3-DJF SST correlation; (H) PC3-MAM SST correlation; and (I) PC3-JJA SST correlation. Positive values mean rising SST leads to increased HWOP frequency and negative values mean rising SST leads to reduced HWOP frequency. Black dots denote areas with significant correlation ($P < 0.05$).

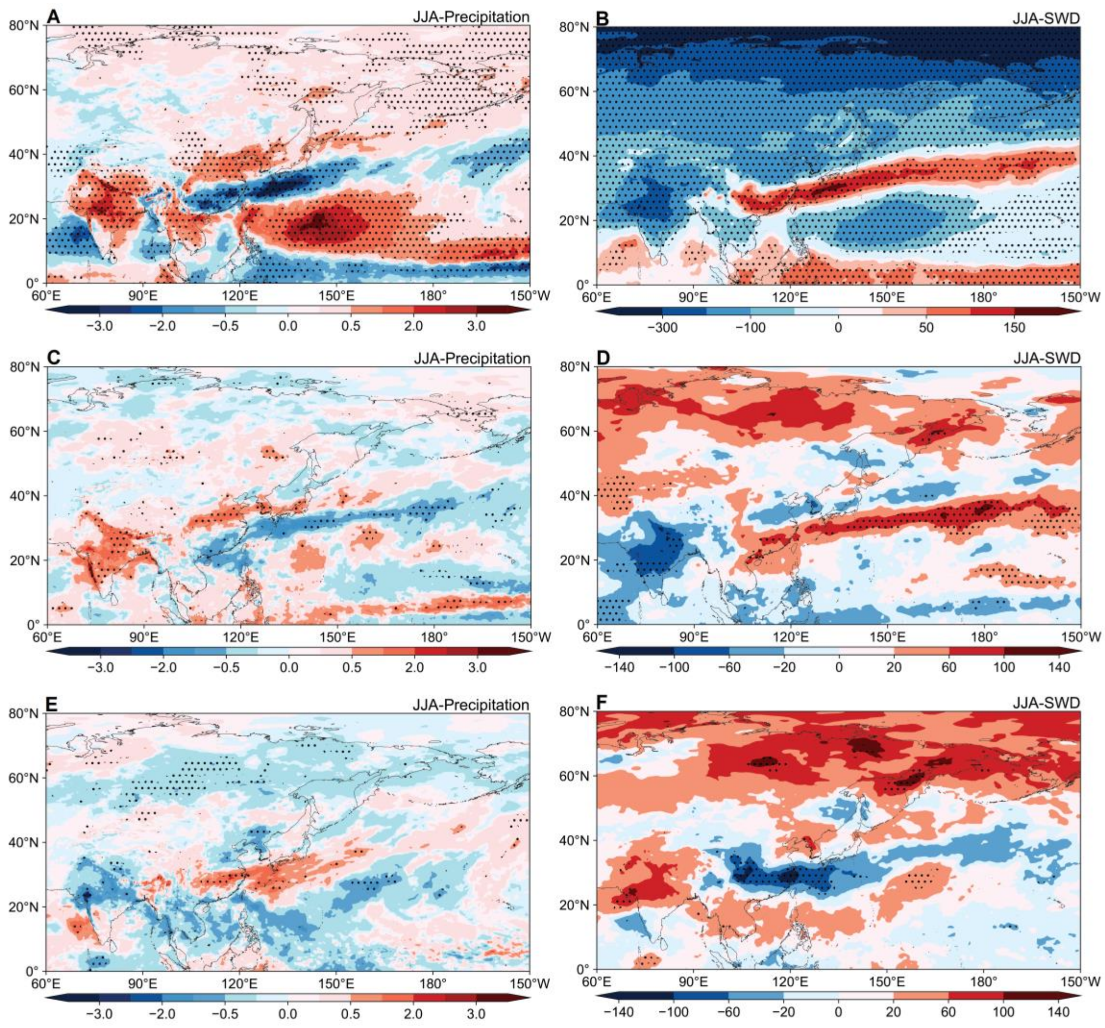

Fig. S7. Regression of precipitation and downward shortwave radiation (SWD) on the first three modes. Precipitation anomalies regressed on (A) PC1, (C) PC2, and (E) PC3. SWD anomalies regressed on (B) PC1, (D) PC2, and (F) PC3. Black dots denote areas with significant correlation ($P < 0.05$).

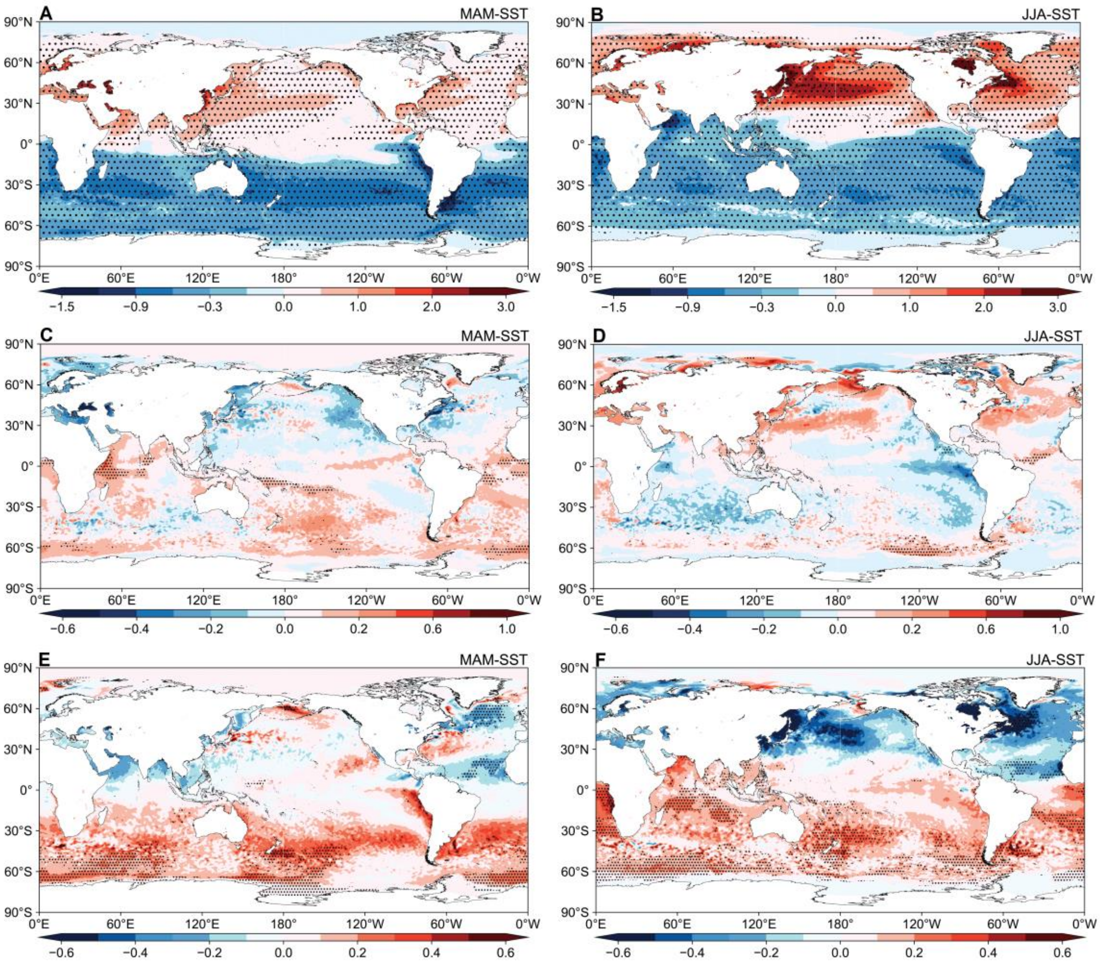

Fig. S8. Regression of sea surface temperature (SST) on the first three modes. MAM SST anomalies regressed on (A) PC1, (C) PC2, and (E) PC3. JJA SST anomalies regressed on (B) PC1, (D) PC2, and (F) PC3. Black dots denote areas with significant correlation ($P < 0.05$).

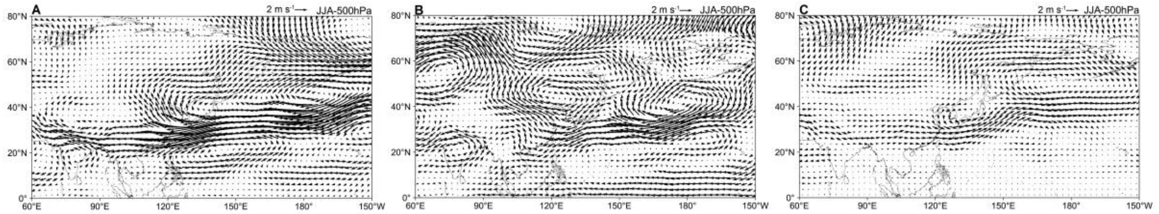

Fig. S9. Regression of 500hPa winds on the first three modes. Wind anomalies regressed on (A) PC1, (B) PC2, and (C) PC3.

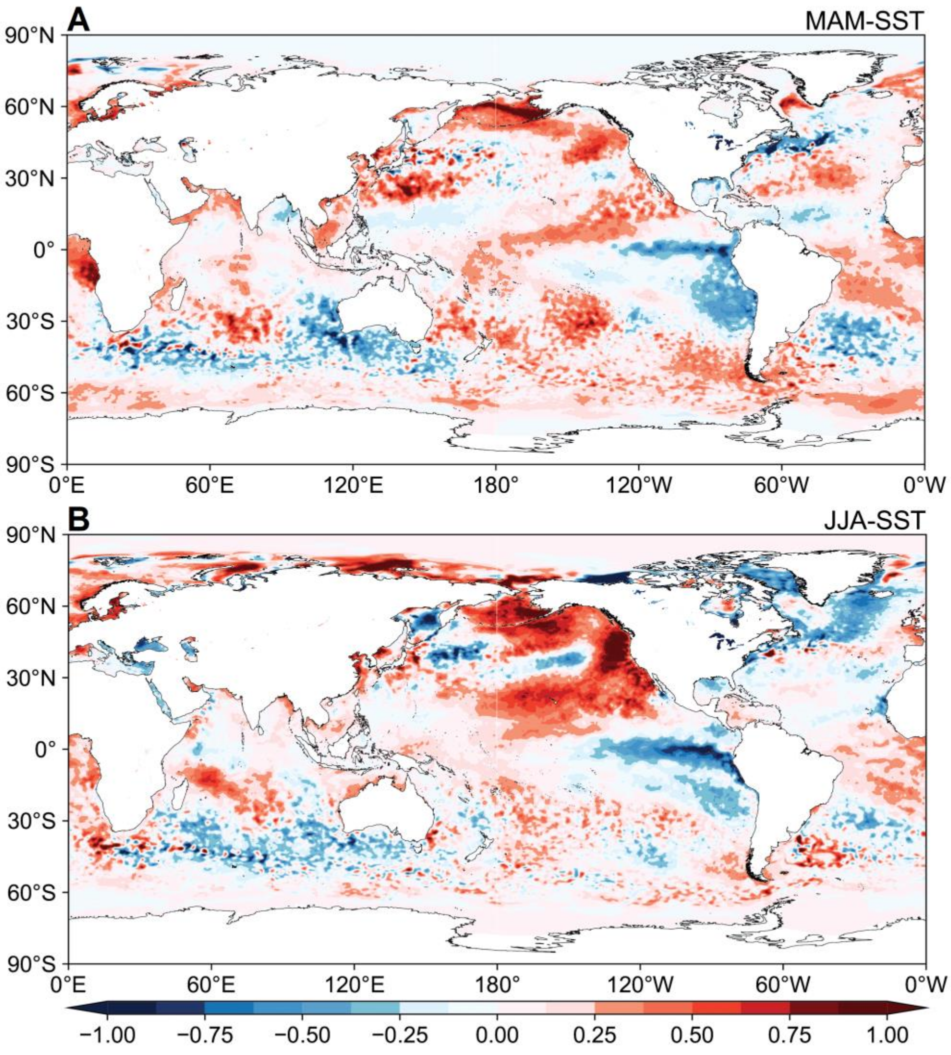

Fig. S10. Composites of sea surface temperature (SST). The composite differences of SST in (A) MAM and (B) JJA between high and low HWOP frequencies.

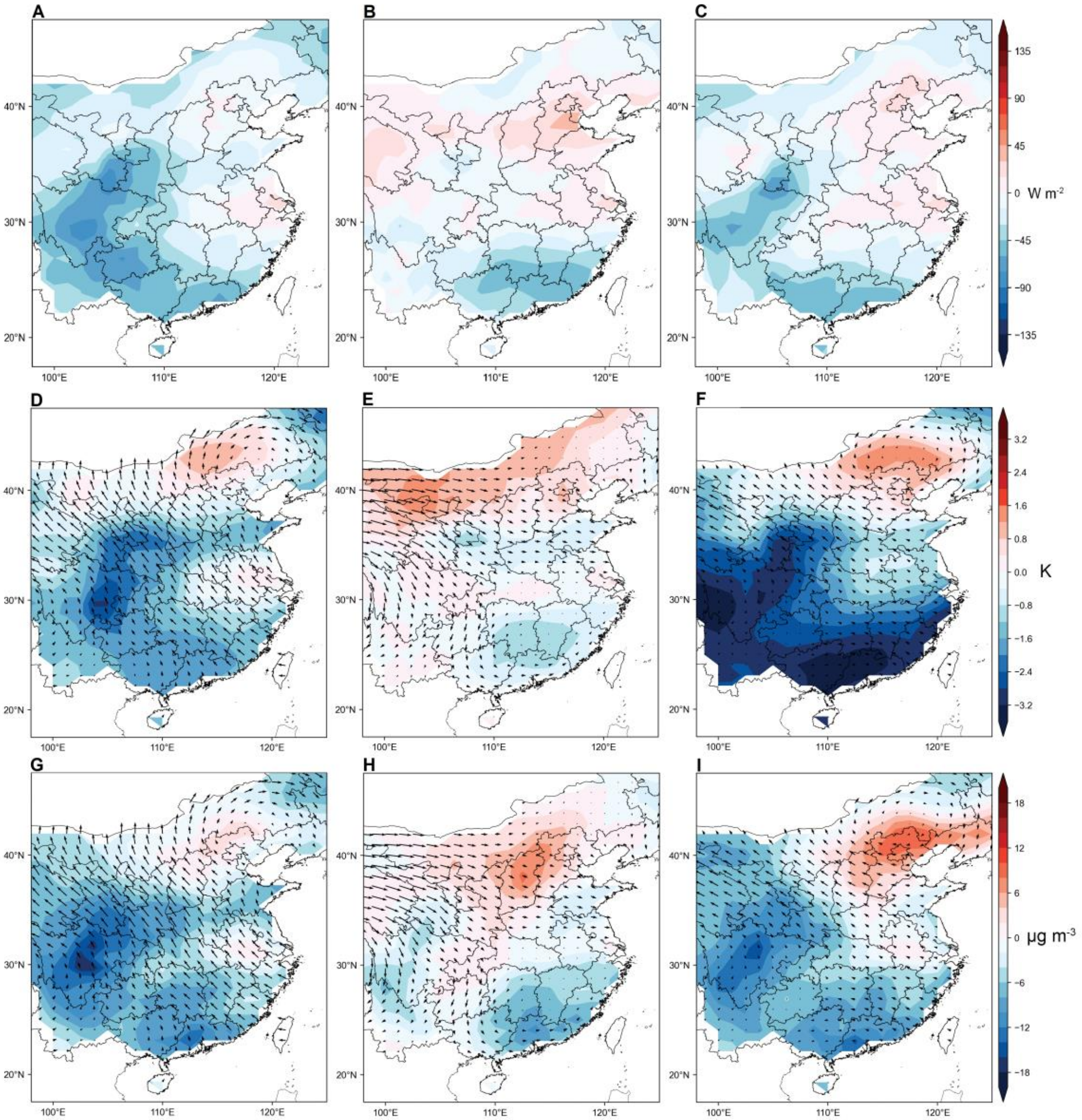

Fig. S11. CESM simulated responses of downward shortwave radiation (SWD), air temperature and O_3 concentration. CESM simulated responses of SWD to springtime (A) SSTwp anomaly, (B) SSTwi anomaly, and (C) SSTRoss anomaly. CESM simulated responses of air temperature to springtime (D) SSTwp anomaly, (E) SSTwi anomaly, and (F) SSTRoss anomaly. CESM simulated responses of O3 concentrations to springtime (G) SSTwp anomaly, (H) SSTwi anomaly, and (I) SSTRoss anomaly.

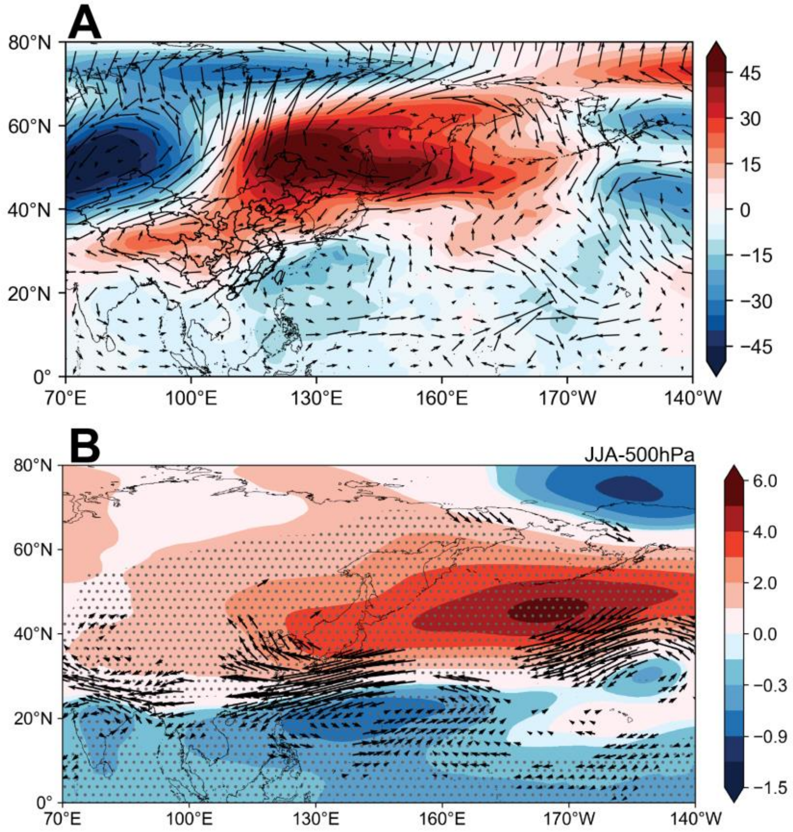

Fig. S12. CESM simulated (A) and observed (B) responses of geopotential height and wind field at 500 hPa to SST anomalies on PC1.

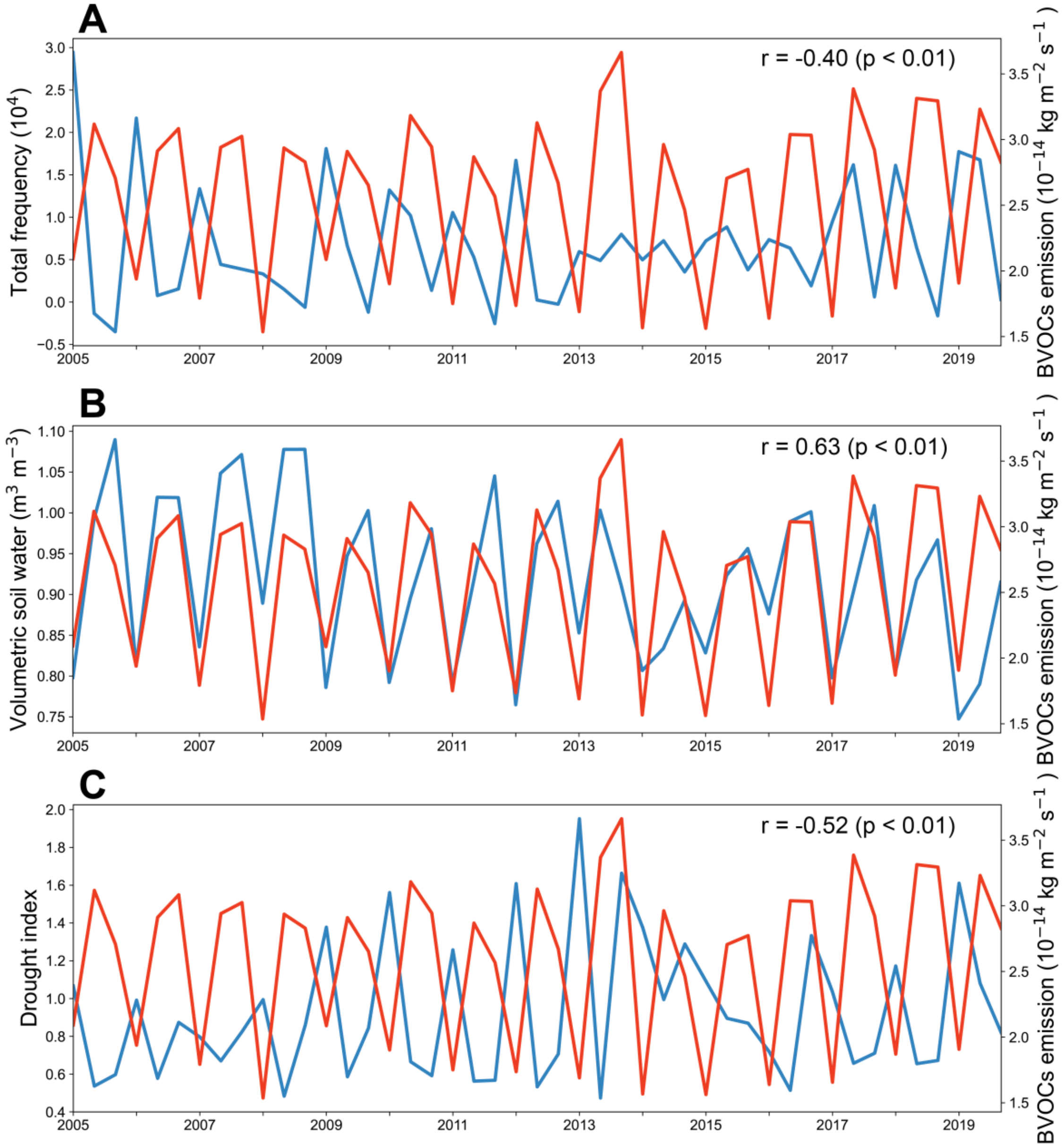

Fig. S13. Variations of HWOP, soil water, drought index and emissions of BVOCs over the NCP. Variations of HWOP in blue line and emissions of BVOCs in red (A). Variations of soil water in blue and emissions of BVOCs in red (B). Variations of drought index in blue and emissions of BVOCs in red (C). Emissions of BVOCs were downloaded from Sindelarova et al. (2022).

#Sindelarova, K., Markova, J., Simpson, D., Huszar, P., Karlicky, J., Darras, S., and Granier, C.: High-resolution biogenic global emission inventory for the time period 2000–2019 for air quality modelling, Earth Syst. Sci. Data, 14, 251–270, https://doi.org/10.5194/essd-14-251-2022, 2022.

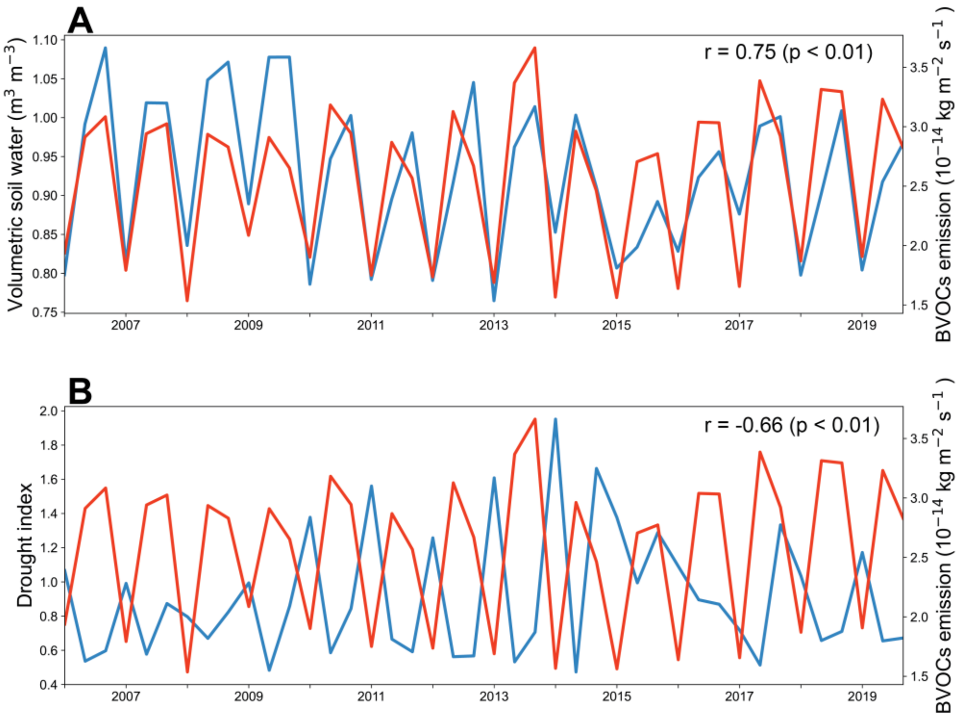

Fig. S14. Variations of emissions of BVOCs, and soil water and drought index in previous years over the NCP. Variations of emissions of BVOCs in red and previous year's soil water in blue (A). Variations of emissions of BVOCs in red and previous year's drought index in blue (B).

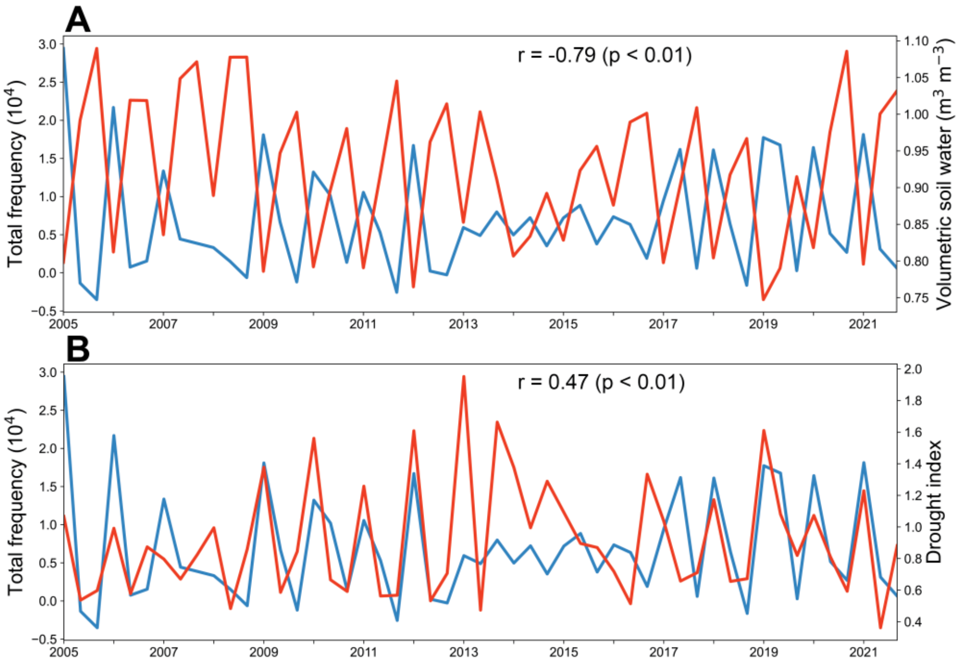

Fig. S15. Variations of HWOP, soil water and drought index over the NCP. Variations of HWOP in blue and soil water in red (A). Variations of HWOP in blue and drought index in red (B).

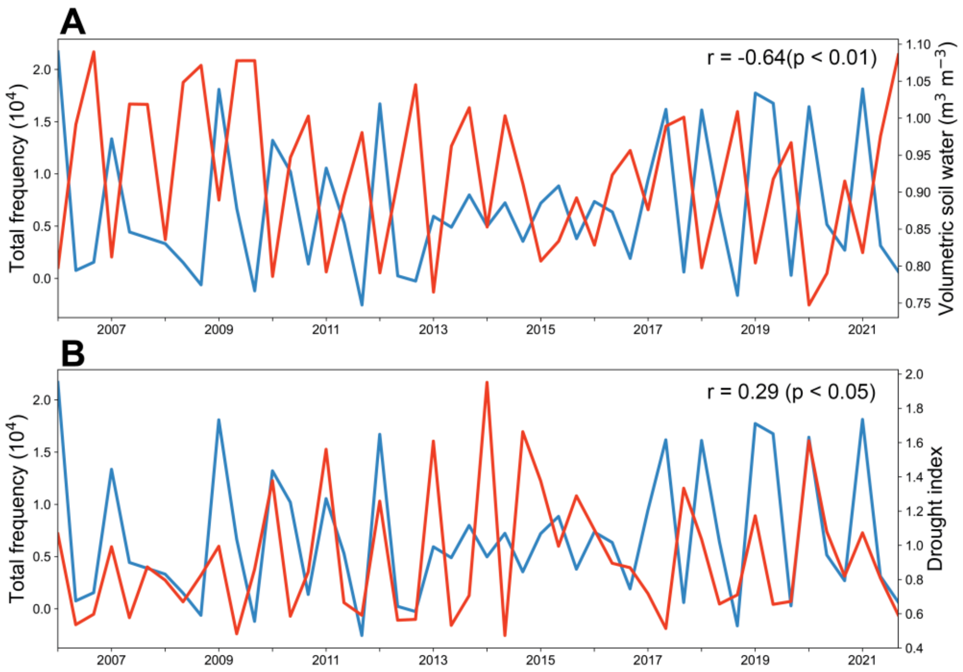

Fig. S16. Variations of HWOP, and soil water and drought index in previous year over the NCP. Variations of HWOP in blue and previous year's soil water in red (A). Variations of HWOP in blue and previous year's drought index in red (B).

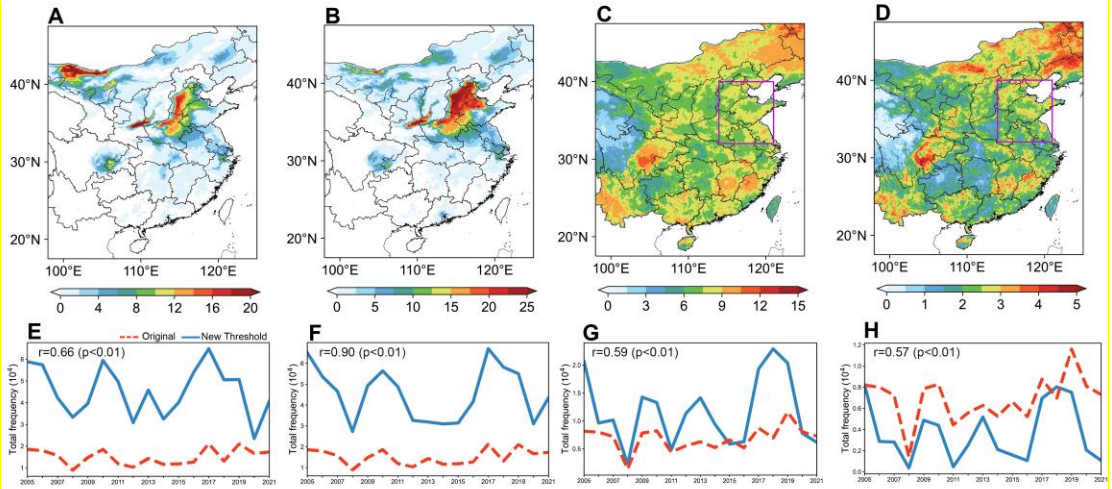

Fig. S17. Spatial distribution and temporal variation of HWOP frequency in summer (days/year) over 2005-2021 in Central and Eastern China based on different thresholds. (A, E) Daily maximum T_{2m} exceeds 35 °C for at least three consecutive days and daily MDA8 O_3 concentrations exceeded 140 μg m^{-3}; (B, F) 33 °C for T_{2m} and 160 μg m^{-3} for MDA8 O_3 concentrations; (C, G) Daily maximum T_{2m} exceeds 80th percentile of 2005-2021 for at least three consecutive days and daily MDA8 O_3 concentrations exceeded 80th percentile of 2005-2021. (D, H) Daily maximum T_{2m} exceeds 90th percentile of 2005-2021 for at least three consecutive days and daily MDA8 O_3 concentrations exceeded 90th percentile of 2005-2021. HWOP shown in (C, G) are for the NCP region.

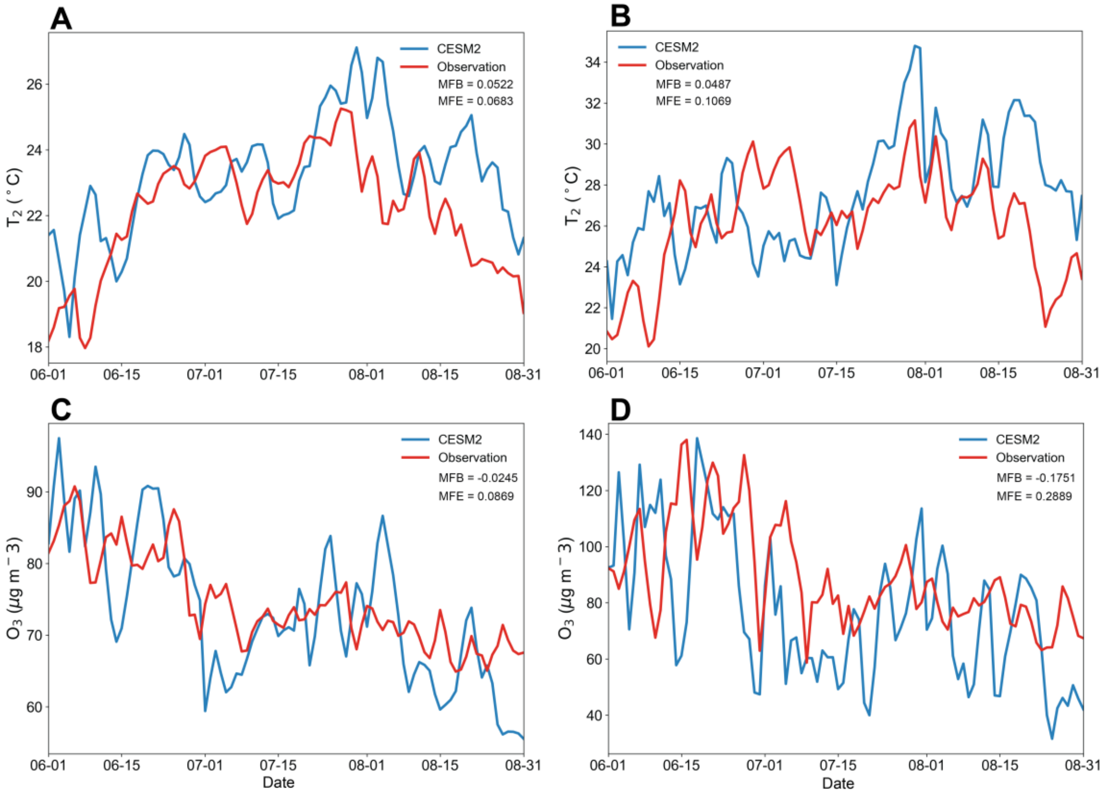

Fig. S18. Model evaluation of the CESM control simulation. Simulated and observed daily (A) surface air temperature in Central and Eastern China, (B) surface air temperature in the NCP, (C) surface ozone concentration in Central and Eastern China, and (D) surface ozone concentration in the NCP. The mean fractional biases (MFBs) and the mean fractional errors (MFEs) of all simulations meet the model performance criteria of within ± 0.6 for MFB and less than + 0.75 for MFE (Boylan and Russell, 2006). Areas of Central and Eastern China and NCP are defined in Fig. 1.

Table S1. AIC values for different combinations of predictors.

Combinations of predictors	AIC values
SST_{wp}	349.88
SST_{India}	342.18
IOD	349.61
SST_{Ross}	343.81
SST_{wp} + SST_{India}	344.17
SST_{wp} + IOD	351.44
SST_{wp} + SST_{Ross}	345.80
SST_{India} + IOD	343.70
SST_{India} + SST_{Ross}	342.04
IOD + SST_{Ross}	345.63
SST_{wp} + SST_{India} + IOD	345.67
SST_{wp} + SST_{India} + SST_{Ross}	339.50
SST_{wp} + IOD + SST_{Ross}	347.62
SST_{India} + IOD + SST_{Ross}	339.89
SST_{wp} + SST_{India} + IOD+ SST_{Ross}	338.30
